# Electrical stimulation methods and protocols for the treatment of traumatic brain injury: a critical review of preclinical research

**DOI:** 10.1186/s12984-023-01159-y

**Published:** 2023-04-25

**Authors:** D. Ziesel, M. Nowakowska, S. Scheruebel, K. Kornmueller, U. Schäfer, R. Schindl, C. Baumgartner, M. Üçal, T. Rienmüller

**Affiliations:** 1 Institute of Health Care Engineering with European Testing Center of Medical Devices, Graz University of Technology, Graz, Austria; 2 Research Unit of Experimental Neurotraumatology, Department of Neurosurgery, Medical University of Graz, Graz, Austria; 3 Gottfried Schatz Research Center for Cell Signaling, Metabolism and Aging, Biophysics Division, Medical University of Graz, Graz, Austria; 4 BioTechMed-Graz, Graz, Austria

**Keywords:** Traumatic brain injury, Transcranial magnetic stimulation, Transcranial direct current stimulation, Deep brain stimulation, Vagus nerve stimulation, Animal models, Recovery, TBI sequelae

## Abstract

**Background:**

Traumatic brain injury (TBI) is a leading cause of disabilities resulting from cognitive and neurological deficits, as well as psychological disorders. Only recently, preclinical research on electrical stimulation methods as a potential treatment of TBI sequelae has gained more traction. However, the underlying mechanisms of the anticipated improvements induced by these methods are still not fully understood. It remains unclear in which stage after TBI they are best applied to optimize the therapeutic outcome, preferably with persisting effects. Studies with animal models address these questions and investigate beneficial long- and short-term changes mediated by these novel modalities.

**Methods:**

In this review, we present the state-of-the-art in preclinical research on electrical stimulation methods used to treat TBI sequelae. We analyze publications on the most commonly used electrical stimulation methods, namely transcranial magnetic stimulation (TMS), transcranial direct current stimulation (tDCS), deep brain stimulation (DBS) and vagus nerve stimulation (VNS), that aim to treat disabilities caused by TBI. We discuss applied stimulation parameters, such as the amplitude, frequency, and length of stimulation, as well as stimulation time frames, specifically the onset of stimulation, how often stimulation sessions were repeated and the total length of the treatment. These parameters are then analyzed in the context of injury severity, the disability under investigation and the stimulated location, and the resulting therapeutic effects are compared. We provide a comprehensive and critical review and discuss directions for future research.

**Results and conclusion:**

We find that the parameters used in studies on each of these stimulation methods vary widely, making it difficult to draw direct comparisons between stimulation protocols and therapeutic outcome. Persisting beneficial effects and adverse consequences of electrical simulation are rarely investigated, leaving many questions about their suitability for clinical applications. Nevertheless, we conclude that the stimulation methods discussed here show promising results that could be further supported by additional research in this field.

## Background

Most recent epidemiological surveillance reports indicate around 223,000 traumatic brain injury related hospitalizations in 2019 and more than 64,000 TBI-related deaths in 2020 in the USA alone [1]. Recent analysis of data from the European Union in 2017 shows a much higher number of TBI-related hospitalizations, although there are less TBI-related deaths [2]. Despite substantial differences among countries, TBI remains a leading cause of mortality and morbidity, particularly amongst the younger population. Decades-long accumulation of clinical and experimental data has set the path to considerable achievements in the clinical management of TBI, which brought a remarkable and gradual reduction in mortality due to head injury [3, 4]. Nevertheless, neurological deficits, cognitive and motor impairments, psychiatric disorders or other morbidities remain among the major sequelae of TBI [5]. Whilst these disabilities render TBI survivors dependent on assistance for daily activities, they also cause severe psychological and economic burden on families due to lifelong patient care.

A modest list of major pathophysiological changes after TBI includes dysregulated cerebral blood flow [6] and impaired cerebral oxygenation leading to ischemic insult [7], glutamate excitotoxicity [8, 9], blood brain barrier breakdown [10], cerebral edema [11, 12], oxidative and nitrosative stress [13, 14], cerebral inflammation [15, 16], hypo- and hyper perfusion [17], mitochondrial dysfunction [18], hemorrhage [19] and hyperemia [20]. The cascade of these pathophysiological changes starts within minutes to hours and days following the primary injury, and may directly or indirectly induce secondary damage to brain tissue, resulting in impaired connectivity and a delayed loss of neuronal cells. Moreover, chronic microglial activation and axonal damage may persist over much longer periods, leading to connectivity loss even years after trauma [21]. Based on the order of appearance of those pathologies, the post-TBI period can be roughly divided into the acute phase lasting minutes to hours after trauma, the subacute phase that lasts several days and is connected to the beginning of the secondary injury, and the chronic phase covering the weeks, months or even years following TBI [22, 23]. Decades of immense clinical and preclinical research were dedicated to deciphering the mechanisms of secondary damage and cell loss. Nevertheless, the continuously increasing knowledge in this field has not yet yielded the desired clinical applications for targeted pharmacological therapies to prevent or attenuate these mechanisms and stop further progression of tissue damage.

Neuromodulation by means of electrical and magnetic stimulation has been used to promote neuroplasticity and connectivity. Although the limited capabilities of nervous tissue to self-repair hinders complete regeneration of damaged brain tissue, processes involved in neuroplasticity can at least partially restore neuronal connectivity. Promising results observed in preclinical and clinical studies with electrical stimulation provide a good basis for the exploitation of neuroplasticity for functional restoration to alleviate trauma-induced disabilities [24, 25]. Hypo- or hyper-excitability, for instance, provide suitable targets for neuromodulatory interventions such as transcranial magnetic stimulation and deep brain stimulation [26, 27]. Supportive treatment of post-traumatic depression using electrical stimulation has also been subject to an immense interest [28, 29]. Preclinical studies, however, which are required to corroborate findings on underlying mechanisms of electrical stimulation and reveal neurobiological correlates of these stimulation techniques, are disproportionately sparse and appear to have attracted increased interest only over the last decade.

In the first part of this article, we give an overview on what is known about the effects of stimulation on neuronal cells and the state-of-the-art of the most commonly used electrical stimulation methods for therapeutic applications. In the second part we present a critical review of the available literature on preclinical studies using electrical stimulation in animal models of traumatic brain injury. The aims are (1) to assess the efficacy of these stimulation methods as post-TBI treatments in preclinical research across several selected studies, (2) to critically compare stimulation protocols as well as treatment time after traumatic insult and (3) to infer the translational value of the reported outcomes for clinical applications.

## State-of-the-art

### Effects of electrical stimulation on neurons

The excitability of neuronal cells facilitates modulation of their firing activity using external stimulation to enhance or suppress endogenous activity [30]. This modulation can be utilized for therapeutic or diagnostic purposes in several neurological diseases or injuries to nervous tissue [31–34]. To better understand the advantages of therapeutic electrical stimulation following TBI, it is necessary to gain extensive insights into how and to which extent stimulation influences neuronal physiology and morphology.

Artificial electrical stimulation may change the electrical potential of the surrounding extracellular region through anodic as well as cathodic protocols [35–37]. In cathodic stimulation, a negative current pulse is delivered to the extracellular area, which in turn depolarizes the cellular membrane with the aim to elicit an action potential. Anodic stimulation instead hyperpolarizes the region near the site of interest and thus decreases the membrane potential [38]. This results in a flux of positive ions towards the stimulation site from surrounding areas, which leads to a depolarization of the cell membrane further away from the site of stimulation, possibly triggering an action potential at the nearest Ranvier node [39, 40].

The effect of electrical stimulation on the brain depends on the inherent characteristics of the tissue. At the cellular level, it is easier to excite an axon than a soma, and myelinated axons are the most excitable part of the cell [41, 42]. Induced voltages differ between nodes and internodes due to the drastic differences in voltage-gated ion channel density [43, 44]. Activated axons progress the signal antidromically to the soma [45, 46] and orthodromically to the synaptic terminals [47, 48]. Bending, branching and significant changes in the diameter of an axon determine the effective site and threshold of the stimulation [43]. Generally, it is easier to elicit action potentials with negative currents in almost all cell compartments, except for some types of dendrites that are more prone to stimulation with positive currents [43, 49, 50].

#### Long-term potentiation (LTP), long-term depression (LTD) and plasticity

Electrical stimulation deeply influences brain electrophysiology through modulation of neuronal signaling not only in the short-term, but also in facilitating or attenuating long-term modifications on a cellular level [51, 52]. Activity-dependent synaptic plasticity can either strengthen or weaken the development of synapses [53, 54], which is crucial for post-traumatic regeneration and recuperation of high-level cognitive abilities like learning and memory formation, loss of which is a typical outcome of TBI [55, 56]. Long-term potentiation (LTP) and long-term depression (LTD) are highly complex and pivotal processes of synaptic plasticity, which may be heavily modified as a consequence of TBI, possibly leading to severe cognitive impairments [56].

LTP is a form of synaptic enhancement resulting in a lasting facilitation of signal transduction. Classically, LTP is elicited through brief high frequency stimulation [57], although it may also be triggered successfully with theta-burst stimulation protocols [58] or chemical compounds [59]. Initiation of LTP requires the activation of postsynaptic *N*-methyl-d-aspartate (NMDA) receptors with glutamate. Subsequent rapid increase of calcium concentration within the cell initiates multiple metabolic cascades and the modulation of gene transcription, resulting in long-term changes to receptor expression, synaptic vesicle transport and other cytoskeletal interactions [55, 60]. LTD is a process analogous to LTP, but leads to reduction of synaptic efficacy. It is usually induced by low frequency stimulation, leading from low to moderate influx of calcium into the neuron mostly through voltage-gated calcium channels and, to a lesser extent, through the activation of NMDA receptors [60, 61].

During a head trauma, mechanical forces applied to nervous tissue disturb ionic fluxes and the concomitant depolarization [62]. This leads to excessive glutamate release from presynaptic axon terminals in the acute phase of the injury that may result in neuronal hyperexcitability and changes in synaptic plasticity. In general, TBI attenuates synaptic LTP responses, while its effect on LTD may vary [56]. LTP deficits and overall increased neuronal excitability were observed soon after injury in in vivo and ex vivo TBI models [63, 64], while the ability to induce LTD was left unchanged [64]. In a controlled cortical impact model in rats, LTD was enhanced as long as 2 days after the initial injury [65]. Considering all of the above, pertinent electrical stimulation protocols to effectively modulate LTP and LTD could be advantageous in the recovery of physiological neuroplasticity mechanisms and the recuperation of impeded motor and cognitive functions following TBI.

#### Spike timing-dependent plasticity (STDP)

Timing of the activation of presynaptic and postsynaptic cells plays a pivotal role in synaptic plasticity [66, 67]. Spike timing-dependent plasticity (STDP) is considered a biologically plausible model for synaptic modifications occurring in vivo [68, 69] and its occurrence has been reported in several brain regions, such as the corticostriatal pathway [70–72], the barrel cortex [73, 74] and the visual cortex [75, 76]. It is determined by the temporal order of action potential initiations and the narrow time between subsequent action potentials. In general, activation of the presynaptic cell immediately before activation of the postsynaptic cell leads to timing-dependent LTP, while activating the presynaptic neuron immediately after the postsynaptic cell elicits timing-dependent LTD [68, 69]. The time window between these activations needs to be in the order of milliseconds, is specific for each synapse and depends on receptor kinetics, current densities and the release of retrograde messengers such as endocannabinoids [69]. Spontaneous spiking as well as changes in the spike frequency can further modulate the strength of plasticity, e.g. higher frequency stimulation has been described to increase the effect of timing-dependent LTP [69]. STDP was observed in both excitatory and inhibitory neurons and could be further modified by cholinergic, dopaminergic and adrenergic signaling [68], enabling prospective pharmacological modulation. It offers an alternative to frequency-dependent stimulation in clinical settings and has already been deployed in human studies to successfully modulate the force of the long-latency stretch reflex in healthy volunteers [77], while overall lower limb motor output was improved in patients with spinal cord injury [78].

### Electrical stimulation methods

**Fig. 1:**
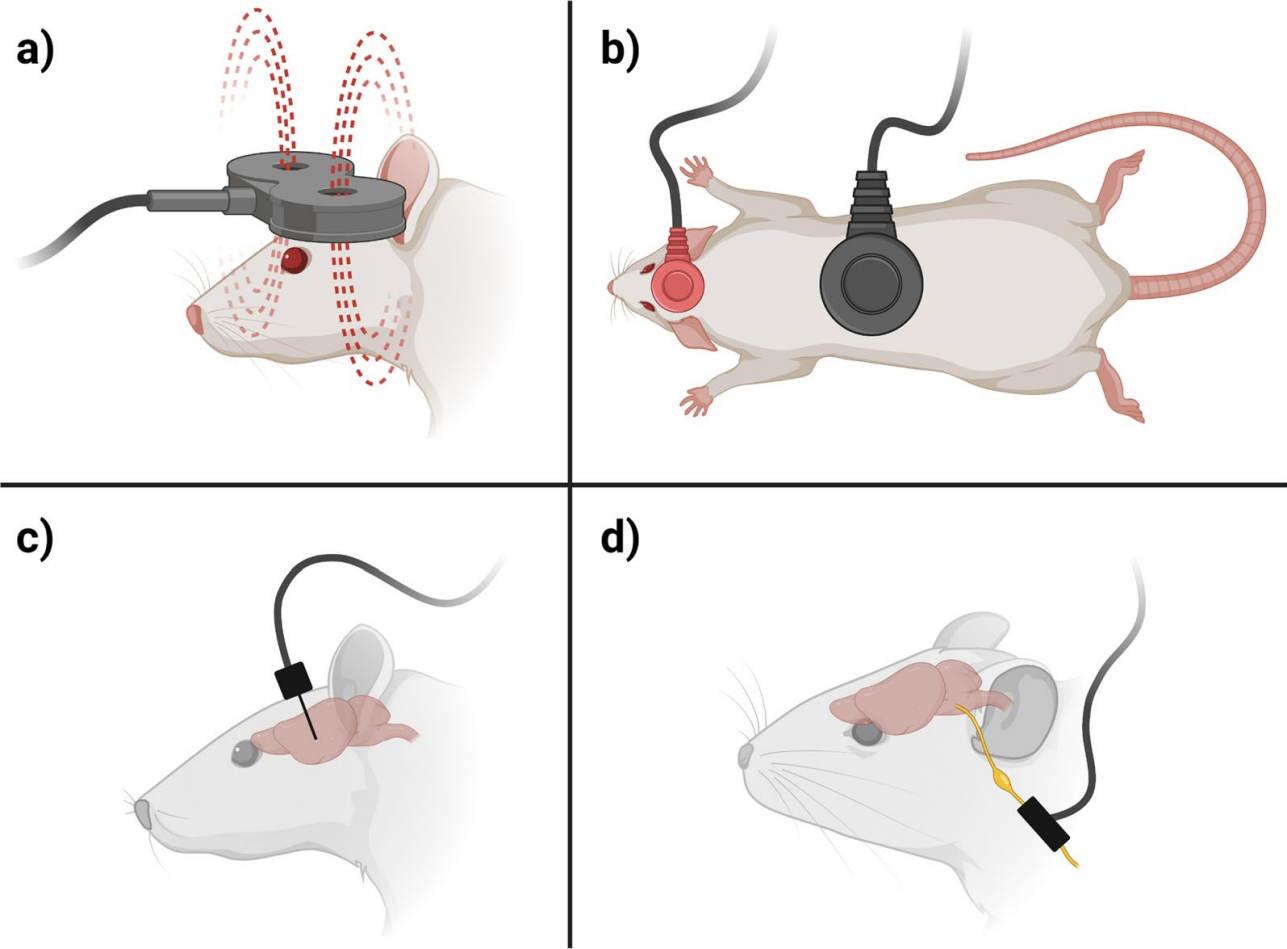
Simplified overview on preclinical applications of the four stimulation methods in the focus of this review: **a** Transcranial magnetic stimulation (TMS) uses magnetic fields to stimulate neurons in the brain non-invasively. **b** Transcranial direct current stimulation (tDCS) delivers low intensity electrical currents to the brain via scalp electrodes in order to modulate neural activity. **c** Deep brain stimulation (DBS) involves the implantation of a device that delivers electrical impulses to specific areas of the brain. **d** Vagus nerve stimulation (VNS) uses cuff electrodes to deliver electrical stimulation to the vagus nerve. Figure created with BioRender.com

The most prevalent electrical stimulation methods used in post-TBI treatment studies, which are in the focus of this review, are transcranial magnetic stimulation (TMS), transcranial direct current stimulation (tDCS), deep brain stimulation (DBS) and vagus nerve stimulation (VNS) [25, 79]. TMS and tDCS are amongst the most commonly used non-invasive brain stimulation techniques [80, 81]. They are effective in the treatment of a wide variety of neurologic impairments, but their efficiency and precision is limited by the distance of the stimulator to the target region. Invasive stimulation methods, such as DBS or VNS, may achieve higher precision and efficiency by bringing the stimulation electrodes closer to the desired area. A schematic overview of these four stimulation methods and their preclinical usage is depicted in Fig. 1.

#### Transcranial magnetic stimulation

TMS is a non-invasive method that utilizes magnetic fields to inhibit or enhance the electrical activity of brain tissue with the aim to improve various neurologic disabilities [82, 83]. This technique utilizes the physical principle of electromagnetic induction by running a high alternating current through a magnetic coil positioned tangentially to the skull of a subject, leading to the formation of a magnetic field that is able to penetrate the skull. When stimulation is applied in the form of pulses, the rapid changes in the magnetic field create electrical currents in the brain, which in turn leads to excitation or inhibition of electrical activity, depending on the frequency of stimulation [84].

The main limitation of this method is that the electromagnetic field created by the coil rapidly decreases in strength with increasing distance. Thus, TMS is mainly used to stimulate cortical areas near the surface of the brain; however, functionally connected regions deeper in the brain can be stimulated indirectly through projecting axons [24]. The depth that the magnetic field penetrates into the brain as well as the size of the stimulated area can be adapted to specific requirements by selecting different coil types with various geometries, materials and designs. Circular coils, for example, can be used to uniformly stimulate a larger volume of neuronal tissue, resulting in greater penetration depth. Figure-of-eight shaped designs, comprising two circular coils positioned next to each other, allow for more selective stimulation at the cost of penetration depth [85]. The area where the two electromagnetic fields produced by this arrangement overlap is characterized by an increased current density compared to the surrounding regions [84]. TMS can be applied in a wide variety of different protocols, most commonly in the form of repetitive pulses, which is usually referred to as repetitive TMS (rTMS) [86].

The therapeutic potential of rTMS is widely recognized, particularly in the field of psychiatry, and it is applied as a treatment option for depression [87, 88] and obsessive–compulsive disorder [89, 90]. Its efficacy was further tested as a treatment for a number of different neurological conditions, such as neuropathic pain [91, 92], epilepsy [93], stroke [94], multiple sclerosis [95] and post-traumatic stress disorder [96], as well as Parkinsonian movement disorders [97, 98].

#### Transcranial direct current stimulation

In contrast to other stimulation methods that employ pulsed protocols for neurostimulation, tDCS uses direct current to influence the cell membranes of neurons in the desired cortical area [99, 100]. A current of several milliamperes is applied via a pad electrode, called the active electrode, attached to the pericranium near the area of interest, which leads to changes in cortical excitability and neuronal activity [101, 102]. A second, larger reference electrode is usually placed further away from the stimulation site. During anodal tDCS, a positive current is applied between the two electrodes, leading to a hyperpolarization of the area near the active electrode, whilst cathodal tDCS depolarizes the tissue with the use of negative currents. The resulting excitation or inhibition of neurons may lead to neuromodulation in affected areas [99, 103]. The current density is crucial for the efficacy and propagation depth of the stimulus [104].

This method is painless, non-invasive and used as a treatment for depression and a variety of cognitive dysfunctions [105, 106]. However, lack of precision is a limiting factor in cases where targeted neurostimulation would be necessary, such as post-traumatic tremor [107].

#### Deep brain stimulation

DBS is an invasive approach that requires the implantation of a stimulation electrode directly into the targeted brain area [108, 109]. The stimulation setup comprises an implanted stimulation electrode and a connected subcutaneous wire that forwards signals from an external stimulating device. Stimulation electrodes are often implanted bilaterally and commonly have multiple metal contacts, which can be used both as anodes and as cathodes [110]. In bipolar configurations, an electrical field is generated between two adjacent contacts, allowing for a concentrated electric field and thus a higher precision [110]. The optimal electrode position is usually determined beforehand with the help of neuroimaging via computed tomography (CT) or magnetic resonance imaging (MRI), which can also be used to guide the surgeon during implantation. Throughout the procedure, electrical activity is continuously measured to ensure correct electrode placement. Afterwards, the efficacy of the implanted device is verified by applying initial stimulation pulses [111].

This method is approved for the symptomatic treatment of Parkinson’s disease, essential tremor, obsessive compulsive disorder and some cases of severe epilepsy in humans [112, 113]. Thanks to its versatility and high spatial resolution, DBS has potential use in the treatment of higher-order cognitive dysfunction and disorders of consciousness in patients with TBI [114].

#### Vagus nerve stimulation

VNS is an indirect brain stimulation method that excites the afferents of the vagus nerve to modulate activity of the central nervous system. While vagal afferents provide sensory information to the brain stem from multiple internal organs, efferents mediate the parasympathetic control of various bodily functions. Thus, VNS results in a wide range of different effects caused by the stimulation of medulla and brainstem including the modulation of neurotransmitters: notably epinephrine, serotonin and gamma-aminobutyric acid [115]. Other potential modes of action include changes in blood flow in several brain regions [116–118], upregulation of neurotrophin production [119], reduction of damage to the blood brain barrier [120–122] and anti-inflammatory effects [123, 124]. VNS systems are approved for treatment of drug-resistant epilepsy [125] and severe, recurrent unipolar and bipolar depression [126], both of which are common disorders developing as a consequence of TBI [127–129].

Most commonly, VNS is used as an invasive modality, employing helical cuff electrodes in monopolar, bipolar or tripolar configurations. These electrodes are usually wrapped around the left cervical vagus nerve [130] to indirectly stimulate distant brain regions. Stimulation of the right vagus nerve might lead to severe bradycardia and is therefore generally avoided [130]. Monopolar electrodes are comparatively cheap, but require an additional ground electrode. Bipolar configurations allow the induced current to flow between two electrodes, enabling a much greater control of the current path. Tripolar electrodes are more expensive, but have the advantage of preventing leakage currents to the surrounding tissue since the stimulating electrode is positioned between two common counter-electrodes.

#### Stimulation waveforms and protocols

The selection of suitable protocols is an important factor for efficacious stimulation, but also for the prevention of damage to the stimulating electrodes and the surrounding tissue [35]. This is particularly relevant for invasive approaches, such as DBS and VNS, where implanted electrodes need to last for longer periods of time and are in direct contact with neural tissue [131]. Unwanted electrochemical reactions at the electrode-tissue interface include corrosion and oxygen reduction reactions, which can be minimized by selecting appropriate stimulation protocols and waveforms [132, 133]. While monophasic pulses are more efficacious for stimulation than biphasic pulses, they potentially result in greater tissue damage, since all injected charge creates electrochemical reaction products and result in greater negative overpotentials over time [35, 134]. Biphasic stimulation, on the other hand, has the potential to reverse electrochemical processes at the electrode-tissue interface, but may also reverse some of the desired effects necessary for efficacious charge induction. Introducing a short interphase delay reduces the suppressing effect of the reversal phase, as long as the delay is short enough to prevent excessive accumulation of electrochemical reaction products [35].

Another important part of the stimulation protocol is the timing of the treatment application after injury, which depends on the selected treatment modality, the severity of the trauma and the goal of the treatment [135]. The onset of stimulation in preclinical studies varies from immediately to several weeks after trauma [25]. In clinical settings, these techniques are usually applied at later stages as a support to traditional rehabilitation methods for treating disabilities that persist after TBI [114, 136].

#### Additional stimulation methods

In addition to the methods mentioned above, there are several other promising electrical stimulation modalities that may be effective in the treatment of TBI sequelae. Electrical cortical stimulation, an invasive method where electrodes are implanted near the cortical surface, can be used to modulate brain plasticity to treat sensorimotor and cognitive deficits in rats [137]. Similarly, epidural electrical stimulation utilizes pulsed stimulation protocols applied to electrodes implanted in the epidural or subdural space to enhance motor recovery and brain activity [138–140]. Promising non-invasive TBI treatment methods include electroconvulsive therapy, which finds use as the treatment for mood disorders such as depression [141], but has not yet been investigated in preclinical TBI models.

Temporal interference stimulation is another novel treatment modality that can be used to stimulate deep brain regions non-invasively, exploiting a well-known acoustic phenomenon [142]. By applying two sinusoidal stimuli in the kilohertz-range with slightly differing frequencies through electrode pairs placed on the head of a patient, interference patterns can be generated inside the brain [143]. The effect of stimuli in the kilohertz range on the underlying tissue is only small due to the filtering properties of cellular membranes [144, 145], and the amplitude of the individual signals is comparably low. Constructive interference of these two signals in the target area leads to an electric field oscillating with an envelope frequency equal to the difference between the two individual signal frequencies. This method has successfully been applied to mouse motor cortex, leading to the elicitation of movements [146].

It is also possible to implant passive components in the brain that convert an external impulse from a source outside the skull into an electrical stimulus. An example for this would be photocapacitors [147–149], which charge up when they are irradiated by light pulses, creating an electric field at their surface, leading to the depolarization of adjacent neural cells. These photocapacitive devices can also be used in combination with temporal interference stimulation protocols [150].

## Systematic literature review

**Fig. 2:**
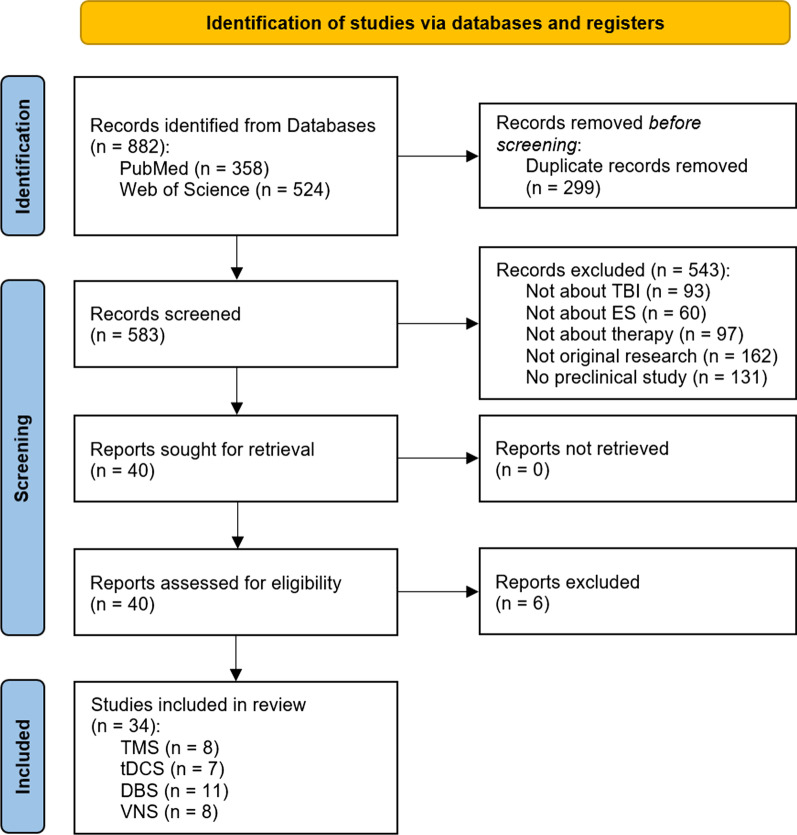
PRISMA 2020 flow diagram depicting the selection process of the studies for this review [151] (*TBI* traumatic brain injury, *ES* electrical stimulation, *TMS* transcranial magnetic stimulation, *tDCS* transcranial direct current stimulation, *DBS* deep brain stimulation, *VNS* vagus nerve stimulation)

To gain further insight into the methods and protocols used for TBI therapy in preclinical studies, an extensive systematic literature search was conducted. The articles included in this survey were found in PubMed and Web of Science. The scientific integrity of the review was ensured by closely following the PRISMA 2020 guidelines [151]. A flow diagram detailing the literature assessment process is given in Fig. 2.

### Search terms: literature identification

To cover the most commonly used variations that describe the stimulation methods selected for this review as well as TBI, the search query consisted of the following MeSH terms:

(“transcranial magnetic stimulation” OR “transcranial direct current stimulation” OR “deep brain stimulation” OR “vagus nerve stimulation” OR "vagal nerve stimulation") AND (“traumatic brain injury” OR “tbi” OR “concussion”)

The search was conducted in the PubMed and Web of Science databases. To obtain as many relevant records as possible, the query was searched in all fields of the respective databases, which includes titles, abstracts and keywords of publications, among other information. The list of search results was last updated on the 8th of September 2022 and the search yielded 358 results in PubMed and 524 in Web of Science, amounting to a total of 583 different records after removing duplicates. The results were sorted by publication date from oldest to most recent and the titles, authors and publication years of these records were exported from the respective databases and collected in a Microsoft Excel spreadsheet to organize further screening.

### Inclusion criteria: literature screening

The abstracts of all 583 individual search results were screened by one investigator for five different criteria of interest to this review. This was done manually without the use of any advanced automation tools except for a simple text search function. First, the abstract needed to mention TBI as the underlying cause of the disability under investigation. Next, an electrical stimulation method had to be utilized in the study and third it had to be used for a therapeutic purpose or as a treatment, as opposed to a diagnostic application. The record also needed to consist of original research, which excluded other review articles and excerpts from larger studies, such as meeting abstracts and conference papers. Finally, only preclinical studies were included, where an animal model was utilized to investigate certain parameters of interest.

These five criteria were assessed in the order described above, and when an article did not contain that criterion, it was immediately excluded from the review. A total of 543 records were excluded, 93 of which did not investigate TBI sequelae, 60 were not about electrical stimulation, 97 used these methods for an application other than therapy, 162 were not original research and 131 of the remaining articles were not preclinical studies. This abstract screening resulted in 40 articles for the following full-text assessment step.

### Eligibility: full-text assessment

Out of the 40 articles selected for full-text assessment, another six were excluded. Four of the excluded articles used electrical stimulation not for the therapy but for the assessment of stimulation effects on healthy animals. One study was not original research, which was not immediately apparent in the abstract, and another article did not utilize electrical stimulation altogether. Ultimately, literature screening led to a total of 34 articles that were reviewed in this study. Eight separate studies used TMS and VNS respectively, seven employed tDCS, and eleven utilized DBS for the treatment of TBI sequelae.

## Results

During full-text assessment, multiple parameters were collected from the 34 selected articles for further analysis and comparison. The first two columns list general information about the respective study, such as its main focus and the impairment under investigation. The next column describes the animal model used in each study, which includes the number and type of animals, the applied TBI model, and if animals were anesthetized during stimulation. After that, the technical aspects of the applied stimulation are summarized, such as the stimulation protocol that was used, the time frame of the stimulation, and the location that was stimulated. The last set of parameters focuses on the assessment of the study results, namely the tests that were conducted with the animals, the parameters that were studied, if they observed any long-term effects of stimulation, and a short summary of the main findings of the paper. All this information was collected in four individual tables, one each for TMS, tDCS, DBS and VNS, which are displayed below (Tables 1, 2, 3, 4).

**Table 1:** Overview of preclinical transcranial magnetic stimulation (TMS) studies

References	Main focus	Impairment	Animal model	Stimulation protocol	Stimulation time frame
Yoon YS et al. [138]	Effects of rTMS and EES on TBI	Motor function	51 male Sprague–Dawley rats (21 died from TBI), Marmarou’s weight drop (450 g from 1 m, diffuse, mild TBI, medial impact), awake and immobilized during TMS	90% of max. device output, 10 Hz, 3 s stim and 6 s pause, for 10 min	Twice per day, day 1–14 post-injury
Yoon KJ et al. [152]	rTMS for behavioral recovery	Motor function, brain metabolism, cell death	20 adult male Sprague–Dawley rats, lateral FPI (3.5–4 atm pressure, severe TBI), awake and immobilized during TMS	80% of RMT, 10 Hz, 15 trains of 2 s, 1 s inter-train interval	10 sessions over 2 weeks, beginning on 4th day post-injury
Lu H et al. [153]	rTMS for pediatric TBI	Motor function	26 juvenile Sprague–Dawley rats, CCI over left primary somatosensory cortex (severity unclear), TMS under 2% isoflurane	25% of max. device output, 20 Hz, 9 trains of 100 pulses, 55 s inter-train interval, for 9 min	Twice per week, starting 9 post-injury, for 4 weeks
Lu X et al. [156]	rTMS for neuromodulation and neurogenesis	Loss of brain parenchyma, reduced brain metabolism, neurological impairment	38 adult Sprague–Dawley rats, Feeney’s weight drop (moderate TBI, right hemisphere), awake and immobilized during TMS	60% of max. device output, 5 Hz, 36 trains of 25 pulses, 15 s inter-train interval, 900 pulses/day, figure-of-eight coil	From 2 days post-injury until 1 day before sacrificed (7/14/28 days after TBI)
Verdugo-Diaz et al. [157]	Treatment with intermediate frequency rTMS	Mortality, general behavioral changes	97 male Wistar rats, Marmarou’s weight drop (motor cortex, severe TBI), awake and immobilized during TMS (animals trained for immobilization)	50% of max. device output (120% of RMT), 2 Hz, 15 min per day, figure-of-eight coil	Starting 1 day post-injury, for 7 consecutive days
Shin et al. [154]	Therapy with rTMS and environmental enrichment	motor function	97 male Sprague–Dawley rats, CCI (4 m/s, moderate TBI, right hemisphere), MEP assessment under isoflurane, electrophysiological recordings under urethane, fMRI under sedation, rTMS under 2% isoflurane	10 Hz, 7 cycles of 4 s, 26 s between cycles, figure-of-eight coil, (stim. intensity unclear)	Starting 1 day post-injury, daily, for 6 days
Sekar et al. [155]	Low-field magnetic stimulation (LFMS, rTMS variant) treatment after TBI	Cognitive and motor functions	48 male C57BL/6 mice, weight drop (60 g from 1 m, closed head trauma, repetitive TBI, once daily for 3 consecutive days, severity unclear), awake and immobilized during TMS	40 Hz, 6 ms pulses, 80 trains of 2 s, 8 s pause, magn. field changes between uniform and linear gradient every 2 min, for 20 min	Once per day, following recovery from rightening reflex after TBI, for 3 days and once on day 4
Qian et al. [158]	Investigation of cellular mechanisms caused by rTMS treatment	General overview	45 male Sprague–Dawley rats, Feeney's weight drop (20 g × 30 cm impact force, moderate TBI), awake and immobilized during rTMS	30% of motor threshold, 40 Hz, 40 trains of 1 s, in 15 s intervals	Starting 4 days post-injury, once daily, for 2 weeks, five times per week

References	Stimulus location	Tests	Acquired parameters	Persistent effects	Main findings
Yoon YS et al. [138]	Center of the coil placed above injury site	Limb placement test, SPRT, RRT, immunohistochemistry	Limb placement changes, SPRT success rate, RRT performance time rate, c-Fos expression	Not investigated	TMS and EES resulted in significant improvement in SPRT and accelerated improvement in RRT, with particularly robust effects of EES
Yoon KJ et al. [152]	Area with largest MEP amplitude at the weaker biceps femoris after suprathreshold stim., side not stated (probably ipsilateral)	Rotarod and beam balance tests, brain MRI, magnetic resonance spectroscopy, western blot, immunohistochemistry	Motor coordination, balance ability, intact and lesioned hemispheric volume, brain metabolism, apoptotic signaling	Not investigated	rTMS did not have beneficial effects on motor recovery, enhancement of anti-apoptotic response in perilesional area
Lu H et al. [153]	Contralateral primary sensory region	Extracellular electrophysiological recordings, fMRI, open field test, forelimb and hindlimb reflex test, immunostaining	CaMKII expression (LTP), MUA responses, LFP magnitude, evoked fMRI cortical responses, behavioral tests (physiology and hyperactivity)	Long-lasting increase of excitability in non-injured cortex after 4 weeks of TMS therapy	Significant increases in evoked-fMRI cortical response, evoked synaptic activity, evoked neuronal firing and expression of neuroplasticity markers, decreased hyperactivity in behavioral tests
Lu X et al. [156]	Whole brain influenced by magnetic field (max. stim. over the center of the brain)	Behavioral tests (mNSS evaluation), hematoxylin and eosin staining, immunohistochemistry, PET examination	Behavioral recovery, relative brain parenchyma loss, cell proliferation and neurogenesis, neuron protection, cell apoptosis, metabolic activity	Not investigated	High-frequency rTMS may decrease mortality, mature neuron loss, apoptosis, improve behavioral recovery, cell proliferation and neurogenesis in the SVZ, metabolic activity in the contralateral site was not affected
Verdugo-Diaz et al. [157]	Injury site	Hunter’s 21-point behavioral-neurological scale, histology	Body weight, food intake, post-TBI bleeding and mortality, neurobehavioral score, cellular morphological changes, disruptions in hippocampal tissue architecture	Not investigated	Movement restriction prevents damage caused by TBI, intermediate-frequency rTMS slightly promotes behavioral and histologic recovery after TBI
Shin et al. [154]	Midpoint between lambda and bregma, medial located	Beam walk and challenge ladder tests, electrophysiology, evoked LFP, MEP assessment, fMRI in the contralateral cortex	Beam traversal latency, mean speed and slips from ladder, MEP amplitude, LFP magnitude, fMRI activation maps	Combination of EE and TMS led to benefits in sensorimotor function lasting up to 6 weeks	Combined therapy with TMS and EE after TBI leads to functional improvements, possibly via cortical excitability and reorganization, long-term effects probably due to EE rather than TMS
Sekar et al. [155]	Cortical and subcortical areas	RRT, open field test, novel location recognition test, immunohistochemistry, western blot	Time on rotarod, locomotor activity, cognitive function, PrPc level in plasma, GFAP, NeuN and PrPc protein levels, CLOCK and CRY2 levels	Not investigated	LFMS treatment improved motor and cognitive function in mice after repetitive TBI, restored PrPc level, decreased proteins associated with circadian rhythm, decreased GFAP levels, increased NeuN levels, and showed neuroprotective effects
Qian et al. [158]	Coil placed above ipsilateral side, close to the scalp	mNSS assessment, TEM, immunohistochemistry, western blot, RT-PCR detection	Injury severity, synaptic ultrastructure, protein expression (BDNF, TrkB, NMDAR1, P-CREB, SYN), mRNA expression levels	Not investigated	rTMS may promote recovery of neurological functions in TBI rats through enhanced SYN protein levels to promote synaptic reconstruction and affecting the expression of proteins related to LTP occurrence

**Table 2:** Overview of preclinical transcranial direct current stimulation (tDCS) studies

References	Main focus	Impairment	Animal model	Stimulation protocol	Stimulation time frame
Yoon et al. [159]	Effects of anodal tDCS on behavioral and spatial memory in early stage TBI	Behavioral and spatial memory	36 male Sprague–Dawley rats, lateral FPI (moderate TBI), anesthetized during tDCS	Anodal tDCS, 0.2 mA, (2.82 mA/cm^2^ current density), for 20 min	Once per day, for 5 days, starting 1 or 2 weeks post-injury
Kim and Han [160]	Effects of anodal tDCS on neuroplasticity	Motor and sensory cortical excitability	31 male Sprague–Dawley rats (postnatal day 42), weight drop (175 g from 30 cm, 3 consecutive times, repetitive mild TBI), anesthetized during all procedures and evaluations	Anodal tDCS, 0.2 mA (0.255 mA/cm^2^ current density), for 30 min	Once, directly after TBI
Bragina et al. [161]	Perfusion and tissue oxygenation after anodal tDCS, motor and cognitive neurologic outcome	mCBF and tissue oxygenation, motor function	40 mice, CCI (5 m/s, 2 mm from cortical surface, mild to moderate TBI), awake during tDCS	Repetitive anodal tDCS, 0.1 mA, for 15 min	Over 4 weeks, for 4 consecutive days at 3-day intervals, starting 1 or 3 weeks post-injury
Yu et al. [162]	Effects of tDCS and ECS on motor and cognitive recovery, brain plasticity, spatial learning and memory	Motor and cognitive function	30 male Sprague–Dawley rats, weight drop (moderate TBI), awake during tDCS	Anodal tDCS, 0.1 mA, 50 Hz, 200 µs pulses, for 30 min	Once per day from days 3 to 28 after electrode positioning
Martens et al. [165]	Cathodal tDCS in the treatment of psychiatric-like symptoms after TBI	Impulsivity and attention	20 male Long-Evans rats, bilateral, frontal CCI (severe TBI), anesthetized during tDCS	Cathodal tDCS, 800 µA (0.708 mA/cm^2^), 10 min	Once per day for 7 days (2 h before testing), starting 6 weeks post-injury
Bragina et al. [164]	Effects of anodal tDCS on cerebrovascular reactivity and mCBF regulation	Cerebrovascular reactivity and mCBF	20 mice, CCI (5 m/s, 2 mm from cortical surface, mild to moderate TBI), awake during tDCS	Anodal tDCS, 0.1 mA, for 15 min	Once, 3 weeks post-injury
Park et al. [163]	Anodal tDCS to improve motor function after repetitive mild TBI	Motor function	65 male Sprague–Dawley rats, weight drop (175 g from 30 cm, once daily for 3 days, repetitive mTBI), anesthetized during tDCS	Anodal tDCS, 0.2 mA (0.255 mA/cm^2^), for 30 min	Once, 24 h after last induction of mTBI

References	Stimulus location	Tests	Acquired parameters	Persistent effects	Main findings
Yoon et al. [159]	Anode over perilesional area, cathode on chest	RRT, Barnes maze test, brain MRI, MRS, immunohistochemical analysis	Behavioral ability, spatial memory, lesion volume, brain edema, metabolites, BDNF expression	Beneficial effects visible 1 weeks after stimulation, no sustained effects after 3 weeks	tDCS increases recovery of spatial and memory functions when applied 2 weeks post injury, only improves spatial memory when applied 1 week post-injury
Kim and Han [160]	Anode around left motor cortex, counter electrode on thorax	MEP and SEP test, brain MRI, immunohistochemical analysis	Recovery of righting reflex, MEP latency and amplitude, SEP latency and amplitude, brain volumetric changes, GFAP expression	Immunohistochemistry performed 12 days after stimulation, showed no significant improvements	Single anodal tDCS after rmTBI induces early recovery of consciousness, increases modulation of cortical excitability and promotes transient motor recovery
Bragina et al. [161]	Anode near craniotomy, counter electrode on thorax	Custom-made LSCI, two-photon LSM, RRT, passive avoidance test, Y-maze test, Nissl staining	Regional and microvascular cerebral blood flow, motor deficits, learning, spatial and working memory	Preserved improvement in learning and motor abilities 1 week after stimulation was ended	Anodal tDCS increases brain microvascular blood flow and tissue oxygenation in TBI and sham mouse brain and could contribute to neurologic improvement
Yu et al. [162]	Anode above lesion, cathode at trunk	Rehabilitation training (SPRT, RRT, Y-maze), neurological examination, histology, immunohistochemistry	Success rate of SPRT and Y-maze tests, average rates of RRT, lesion assessments, c-Fos expression	Not investigated	ES with rehabilitation training for TBI rats is effective for motor recovery and brain plasticity, ECS induces faster behavioral and cognitive improvements than tDCS
Martens et al. [165]	Cathode near bregma, anode between scapulae	Five-choice serial reaction time task, analysis of brain slices to verify injury severity	Motor impulsivity, attention, relationship between magnitude of impairment and recovery	No lingering effects observed, disappeared after stimulation stopped	Relationship between magnitude of impulsive deficit and degree of tDCS-recovery, the most severely impaired subjects benefit the most from neuromodulation
Bragina et al. [164]	Anode near craniotomy, cathode on thorax	Two-photon LSM (before and after stimulation), cerebrovascular reactivity test (hypercapnia)	mCBF (arteriolar diameter), brain tissue oxygen flow (NADH autofluorescence)	Not investigated	Anodal tDCS restores cerebrovascular reactivity of parenchymal arterioles and regulation of mCBF, could contribute to neurologic improvement
Park et al. [163]	Anode over left M1 area, cathode on trunk	Brain MRI, histology, MEP evaluation (via TMS and needle electrodes), foot-fault test, rotarod test	Damage evaluation after repetitive mTBI, MEP amplitude and latency, motor coordination, sensorimotor function, balance alterations	Not investigated	Anodal tDCS at the M1 area after repetitive mTBI could improve MEP amplitude, balance control, postural orientation and motor endurance by activating the CST

**Table 3:** Overview of preclinical deep brain stimulation (DBS) studies

References	Main focus	Impairment	Animal model	Stimulation protocol	Stimulation time frame
Lee et al. [166]	Theta frequency DBS to improve spatial memory	Cognitive deficits	56 adult male Sprague–Dawley rats, lateral FPI (moderate TBI), awake during DBS	80 µA, 7.7 Hz, 1 ms pulses, for 1 min in exp. 1 and for 15 min in exp. 2	From post-injury days 5 to 7, directly before Barnes maze experiment
Gonzalez et al. [167]	Behavioral and anatomical recovery after TBI	Cognitive deficits	79 adult male Sprague–Dawley rats, FPI (moderate TBI), awake during DBS	30 µA, 8 or 24 Hz, 1 ms pulses, 5 min alternated with 5 min break, over 12 daylight hours	Starting 4–6 h post-injury (or after 7 days in one group), for 8 weeks
Tabansky et al. [175]	Temporally-patterned DBS after multiple TBI	Decreased arousal	25 C57BL/6J mice (6–9 weeks old), weight drop (20 g from 25 cm, up to 5 times, moderate TBI), awake during DBS	150 µA, 200 µs biphasic pulses, 125 Hz, for 10 min every 4 h over 1 day, diff. temporal patterns (varying interpulse intervals)	Starting 4–6 h post-injury, over the course of 1 day
Lee et al. [168]	DBS to improve cognition after TBI	Cognitive deficits	136 adult male Harlan Sprague–Dawley rats, lateral FPI (moderate TBI), awake during DBS	20/80/200 µA, 7.7/100 Hz, 1 ms pulses exp. 1: for 15/30/60 s; exp. 2 and 3: starting 1 min before task, for 6 min	Exp. 1: 4 and 5 days post-injury, 2x/day; exp. 2 and 3: 5–7 days post-injury, 2x/day
Chan et al. [171]	Motor recovery with DBS	Motor deficits	32 male Long Evans Rats (7 were withdrawn), FPI in motor cortex contralateral to dominant forelimb (severity unclear), awake during DBS	80% of individual motor threshold, 30 Hz, 400 µs pulses, 12 h per day	Starting 4 weeks post-injury, for 4 weeks
Jen et al. [172]	DBS to modulate bladder function in TBI animals	Bladder dysfunction	22 female Sprague–Dawley rats, weight drop (450 g from 2 m, severe TBI), anesthetized during DBS and cystometry	1.5 V, 50 Hz, 182 µs pulses	One session, 1 week post-injury, during cystometry, triggered by EUS-EMG
Praveen Rajneesh et al. [173]	DBS to treat bladder dysfunction after TBI	Bladder dysfunction	49 male Sprague–Dawley rats, weight drop (450 g from 0.5, 1, 1.5, 2 and 2.25 m, severity unclear), anesthetized during DBS and cystometry	1/1.5/2/2.5 V, 50 Hz, 182 µs biphasic pulses, for 10 s	One session, 1 week post-injury, during cystometry when bladder pressure exceeded threshold
Praveen Rajneesh et al. [174]	DBS to improve bladder function after TBI	Bladder dysfunction	28 male Sprague–Dawley rats, weight drop (450 g from 2 m, severe TBI), anesthetized during DBS and cystometry	1/1.5/2/2.5 V (randomized sequence), 50 Hz, 182 µs pulses, for 10 s	One session, 1 week post-injury, during cystometry when bladder pressure exceeded threshold
Dong et al. [176]	DBS to promote wakefulness after TBI	DoC	55 Sprague–Dawley rats (28 male, 27 female), weight drop (400 g dropped from 40 to 44 cm, severity unclear), comatose but without anesthesia during DBS	2–4 V, 200 Hz, 0.1 ms pulses, switch between left and right side of lateral hypothalamus every 5 min, for 1 h	Once, 2 h post-injury (1 h after electrode implantation)
Aronson et al. [169]	Task-matched DBS to improve cognitive recovery after TBI	Cognitive deficits	65 adult male C57BL/6 mice, CCI (5.2 m/s, 2.65 mm depth, moderate TBI), awake during DBS	50 µA, 130 Hz, biphasic pulses, 80 µs per phase, 500 ms trains, 500 ms between trains	Starting 2 weeks post-injury, during Morris water maze, 5 s after success for 5 s, four times per day, for 5 days
Chan et al. [170]	DBS to enhance cognitive recovery after TBI	Cognitive deficits	33 male Long Evans rats, CCI (2.25 m/s, 2.5 mm depth, severity unclear), awake during DBS	80% of motor threshold, 30 Hz, 400 µs pulses, charge-balanced	Starting 8 weeks post-injury, 12 h daily, for 4 weeks

References	Stimulus location	Tests	Acquired parameters	Persistent effects	Main findings
Lee et al. [166]	Medial septal nucleus	Video-EEG, Barnes maze	Exp 1.: electrode placement, spatial working memory, search strategy; exp. 2: hippocampal theta power (during stim. and after 15 min)	No persisting effects observed	FPI attenuates hippocampal theta, MSN theta frequency stimulation immediately before trials improves spatial working memory
Gonzalez et al. [167]	Midbrain median raphe and dorsal raphe	Morris water maze, neuroanatomical analysis, cylinder test	Reference memory, working memory, forelimb reaching asymmetry, forebrain volumes, cAMP levels	Not investigated	8 Hz early MR stimulation can restore forelimb reaching, reference memory, working memory and parietal-occipital cortex volume
Tabansky et al. [175]	Central thalamus (bilaterally)	NSS test (circular open maze, hindlimb reflex, beam walk), parental care, elevated plus maze, light–dark transition, pheromenal spatial learning, T-maze, partition test, social discrimination	Injury severity (NSS) and effects of DBS: motor activity deficits, recovery without intervention, nocturnal behavior pattern, behavioral changes	Not investigated	Multiple TBI results in acute deficits for 11–14 days, chaotic simulation increases motor activity more than fixed or random stimulation
Lee et al. [168]	Medial septal nucleus	EEG, object exploration task, Barnes maze, histology	EEG (theta frequency time, phase coherence, peak frequency), behavioral changes (object exploration, search strategy)	No persisting effects observed	FPI diminishes hippocampal theta, no change in phase coherence, shift in peak frequency, MSN stimulation increased hippocampal theta
Chan et al. [171]	Contralateral LCN	Pasta matrix test, cylinder and horizontal ladder tests, histology, RNA microarray assay, immunohistochemistry, western blot	Forepaw dexterity, spontaneous forepaw use, motor coordination, electrode location, lesion volume, various genetic and cellular parameters	Not investigated	LCN DBS can enhance motor recovery after TBI by elevating neuronal excitability and mediating anti-apoptotic and anti-inflammatory effects
Jen et al. [172]	Rostral pontine reticular nucleus (PnO)	EUS-EMG, continuous-infusion cystometry, MRI, assessment of closed-loop control DBS prototype to improve voiding function	Cystometric parameters (volume threshold, contraction amplitude and duration, residual and voided volume, voiding efficiency), electrode position, tissue damage	Not investigated	Designed DBS closed-loop control system prototype for TBI rats and proved its feasibility (detected bladder voiding cycles, significantly improved voiding efficiency)
Praveen Rajneesh et al. [173]	Rostral pontine reticular nucleus (PnO)	Impact height, cystometric measurements, MRI	Effect of impact height on mortality rate, cystometric parameters (volume threshold, contraction amplitude and duration), TBI impact, electrode position	Not investigated	Established weight drop TBI model for significant voiding dysfunction, show therapeutic effects of PnO-DBS on voiding dysfunction and bladder control in rats after TBI
Praveen Rajneesh et al. [174]	Pedunculopontine tegmental nucleus (PPTg)	Cystometric measurements (CMG), external urethral sphincter electromyography (EUS-EMG), MRI	Cystometric parameters, EUS-EMG parameters (burst period, active period and silent period), DBS electrode tip localization	Not investigated	DBS was capable of inducing potential neural regulation that could control bladder functions, PPTg is a promising target of new therapies for lower urinary tract dysfunction
Dong et al. [176]	Lateral hypothalamic area, left and right side	Assessment of consciousness, OX1R antagonist injection, EEG, western blot analysis, immunohistochemistry	Degree of consciousness (I–VI), delta activity, protein expression (OX1R, α1-AR and GABABR)	Not investigated	LHA-DBS-induced wake promotion results in upregulation of α1-AR expression and downregulation of GABABR expression mediated by the orexins/OX1R pathway, LHA-DBS can be used to promote wakefulness
Aronson et al. [169]	Unilateral, cathode in the nucleus accumbens, anode just below the dura	Morris water maze, real-time place preference assay, immunohistochemistry, gene expression analysis	Spatial memory performance, search pattern efficiency, hedonic response, synaptic density and neuronal growth (synapsin-1 and GAP43), neurogenesis	Persistent effects observed 10 days after stimulation cessation	Task-matched DBS of the nucleus accumbens improves recovery of spatial memory in a TBI mouse model, stimulation led to cellular adaptation and upregulation of genes associated with neural differentiation, migration, cell signaling and proliferation
Chan et al. [170]	LCN, unilateral	Barnes maze, baited Y-maze, novel object recognition task, immunohistochemistry, Western blot, Nissl staining	Long-term spatial memory, memory retention, recognition memory, electrode placement, protein expression (CaMKIIα, BDNF, p75NTR), pre- (synapsin I) and post-synaptic (PSD-95) markers	Not investigated	Unilateral LCN DBS is an effective treatment for cognitive deficits in a TBI rat model by enhancing functional connectivity across perilesional cortical and thalamic brain regions

**Table 4:** Overview of preclinical vagus nerve stimulation (VNS) studies

References	Main focus	Impairment	Animal model	Stimulation protocol	Stimulation time frame
Smith et al. [177]	VNS to increase cognitive and motor recovery after TBI	Motor and cognitive function	57 male Long-Evans hooded rats, lateral FPI (left hemisphere, moderate TBI), awake during VNS	0.5 mA, 20 Hz, 30 s trains of 0.5 ms biphasic pulses, 30 min intervals	Starting 2 h post-injury, for 14 days
Smith et al. [178]	VNS for functional recovery after TBI	Motor and cognitive deficits	48 Long Evans hooded rats, FPI (moderate TBI), awake during VNS	0.5 mA, 20 Hz, 30 s trains of 0.5 ms biphasic pulses, 30 min intervals	Starting 24 h post-injury, for 14 days
Neese et al. [184]	VNS to protect GABAergic neurons after TBI	Reduction of GABAergic neurons	24 male Long Evans hooded rats, unilateral FPI (severity unclear), awake during VNS	0.5 mA, 20 Hz, 30 s trains of 0.5 ms biphasic pulses, 30 min intervals	Starting 24 h post-injury, for 14 days
Clough et al. [182]	Effects of VNS on development of cerebral edema	Cerebral edema	19 male Long Evans hooded rats, unilateral FPI (moderate TBI), awake during VNS	0.5 mA, 20 Hz, 30 s trains of 0.5 ms biphasic pulses, 30 min intervals	Starting 2 h post-injury, for 48 h
Zhou et al. [183]	Neuroprotective effects of VNS	Brain edema	28 adult male New Zealand rabbits, brain explosive injury (firecracker with charge quantity of 50 ± 5 mg black powder, severity unclear), conscious during injury (unclear for VNS)	10 V, 5 Hz, 5 ms pulses, for 20 min	Starting 1 h post-injury, for 20 min
Pruitt et al. [179]	VNS with physical rehabilitation to enhance recovery	Motor function	28 adult female Sprague–Dawley rats, CCI to cortex (3 m/s impact, severity unclear), awake during VNS	0.8 mA, 30 Hz, 500 ms trains of 15 biphasic pulses, 100 µs phase duration	Starting on day 9 post-injury, within 45 ms of successful trials, alongside rehabilitation
Dong and Feng [180]	VNS to promote wakefulness after TBI	DoC	120 Sprague–Dawley rats (half male, half female), weight drop (400 g dropped from 40 to 44 cm, severity unclear), anesthetized during VNS	1 mA, 30 Hz, 0.5 ms pulses, for 15 min	Once, directly after TBI
Dong et al. [181]	VNS for wake-promotion after TBI	DoC	120 male Sprague–Dawley rats, weight drop (400 g dropped from 40 to 44 cm, severity unclear), anesthetized during VNS	1 mA, 30 Hz, 0.5 ms pulse width, for 15 min	Once, directly after TBI

References	Stimulus location	Tests	Acquired parameters	Persistent effects	Main findings
Smith et al. [177]	Left vagus nerve, cervical part	Skilled forelimb reaching, beam walk, inclined plane, forelimb flexion, locomotor placing, Morris water maze, histology	Behavioral recovery, cognitive recovery, histologic changes (lesion cavity size, neurodegeneration, hippocampal pyramidal neuron death, reactive astrocytosis)	Not investigated	VNS improves the rate of recovery and performance of rats in a FPI model as shown in multiple behavioral and cognitive tests
Smith et al. [178]	Left vagus nerve	Injury severity, skilled forelimb reaching, beam walk, forelimb flexion, locomotor placing, Morris water maze, histology	Duration of apnea and unconsciousness, behavioral and cognitive recovery, lesion analysis (tissue loss near injury), neurodegeneration (FluoroJade)	Not investigated	VNS facilitates rate of recovery and final level of motor and cognitive performance following FPI, can be applied starting 2–24 h post-injury
Neese et al. [184]	Left vagus nerve, cervical part	Histology	Number of GAD positive cells in cerebral cortices and hippocampal hilus	Not investigated	FPI induces a significant loss of GAD-like immunoreactive cells, VNS has an overall protective effect on GABAergic neurons
Clough et al. [182]	Left vagus nerve, cervical part	Beam walk, locomotor placing	Vestibulomotor function, motor coordination, coordination of limb placing, regional brain water content	Not investigated	Chronic, intermittent VNS in rats attenuates development of cerebral edema
Zhou et al. [183]	Right vagus nerve	CT imaging, blood analysis, histology	Cranial CT images, TNF-α, IL-1β and IL-10 serum concentrations, histological parameters (pathological manifestations, brain water content)	Not investigated	VNS reduced levels of TNF-α and IL-1β, increased levels of IL-10, and reduced degree of cerebral edema, VNS may exert neuroprotective effects against explosive injury
Pruitt et al. [179]	Left vagus nerve, cervical part	Two 30 min behavioral training sessions (pull task) per day (5 days per week, starting 7 days after VNS implantation, for 6 weeks), histology	Pull task performance, mean maximal pull force, motor recovery, lesion size	Not investigated	VNS paired with physical rehabilitation enhances recovery of forelimb function and pull strength after TBI
Dong and Feng [180]	Left vagus nerve, cervical part	OX1R antagonist injection, assessment of consciousness, ELISA, western blot analysis, immunohistochemistry	Behavior and consciousness levels 1 h after TBI, orexin-A and OX1R expression in prefrontal cortex at 6, 12 and 24 h after TBI	Not investigated	VNS might promote wakefulness in comatose TBI rats through upregulation of orexin-A and OX1R expression in prefrontal cortex, VNS is a promising method to wake patients from TBI-induced coma
Dong et al. [181]	Left vagus nerve, cervical part	OX1R antagonist injection, assessment of consciousness, western blot analysis, immunohistochemistry	Degree of consciousness (I–VI), protein concentration in brain tissue (excitatory and inhibitory neurotransmitter receptors), brain section visualization	Not investigated	VNS could promote arousal and improve consciousness after TBI, potential treatment for comatose individuals affected by TBI

### Transcranial magnetic stimulation

Most of the included TMS studies listed in Table 1 investigated the loss of motor functions after TBI [138, 152–155], while some also used it as a potential treatment for detrimental changes in brain metabolism [152, 156], behavioral impairments [157], and to prevent cell death [152, 156]. A recent study also investigated the mechanisms of rTMS treatment without considering any specific disability [158]. Animals were usually immobilized and awake during stimulation, except for two studies, where TMS was applied during temporary anesthesia using volatile anesthetics [153, 154]. In four of the studies stimulation was done at the ipsilateral side [138, 152, 157, 158], in one at the contralateral side [153], and in two at the medial alignment to the injury site [154, 156]. Stimulus intensities are rarely given as absolute values, such as a magnetic field strength, but as a percentage of the maximum output of the stimulator [138, 153, 156, 157] or of the experimentally determined resting motor threshold of the animal [152, 158], while two studies do not specify the intensity of the stimulation [154, 155]. Many protocols employed rTMS in the form of pulse trains at frequencies ranging from 2 to 40 Hz, some of them having applied the stimulus for 9 to 20 min [138, 153, 155–158], while others stimulated for 3 min or less [152, 154]. In half of the studies TMS treatment was started 1 day after injury [138, 154, 156, 157], and the other half started stimulation several days later [152, 153, 155, 158]. Stimulation sessions were usually administered daily and continued for 1 week or longer. The target of TMS was often a nonspecific area of the cortex, apart from one study where the primary sensory region on the non-injured side of the brain was stimulated in pediatric animals [153] and another study that specifically targeted subcortical areas [155]. Persisting effects of TMS were rarely investigated, but one research group claims to have found a long-lasting increase of excitability in the non-injured cortex [153], while another found functional improvements lasting for up to 6 weeks after stimulation when TMS was combined with environmental enrichment [154]. Three studies observed a neuroprotective effect and the prevention of cell death [152, 155, 156], while two each determined that TMS could be an effective treatment to improve motor function [138, 155], induce neural plasticity [153, 158], or help with the recovery of brain activity [138, 152]. It was also shown that TMS led to histologic improvements after TBI, meaning that the expression levels of relevant proteins changed towards a positive outcome [155, 157, 158]. Individual studies determined that TMS could decrease hyperactivity [153], improve cell metabolism and at the same time induce cell proliferation and neurogenesis [156], help with the recovery from behavioral impairments [157], improve cortical excitability [154], or enhance cognitive function [155]. Only one study did not observe any improvements in motor function after applying TMS [152].

### Transcranial direct current stimulation

The studies shown in Table 2 used tDCS mainly to assess improvements in motor function, excitability and cognitive impairments [159–163], but also its effects on cerebral blood flow (CBF) and tissue oxygenation after TBI [161, 164]. Only one study examined tDCS as a treatment for psychiatric-like symptoms such as impulsivity and attention [165]. Animals were anesthetized during tDCS in four of the seven studies [159, 160, 163, 165] and stimulation was applied for 10–30 min in all studies. In four studies, sessions were repeated for several days and lasted up to 4 weeks [159, 161, 162, 165], while three studies applied the stimulation only once in either the acute [160], subacute [163] or chronic phase [164] after TBI respectively. In six of the seven studies [159–164] anodal tDCS with an amplitude between 0.1 and 0.2 mA was applied. Nevertheless, the surface area of the employed electrodes varied considerably, resulting in widely different current densities between 0.255 and 2.82 mA/cm^2^, which is a critical factor for effective stimulation [104]. The anode was usually placed near the lesion or motor cortex, and the cathode at the thorax or trunk of the animal. Only one study [165] employed cathodal instead of anodal tDCS with a higher amplitude of 0.8 mA, resulting in a current density of 0.708 mA/cm^2^, whereby the cathode was placed near the bregma and the anode between the scapulae. One group observed a persisting increase in local cortical CBF in response to tDCS in TBI and control animals, as well as improved motor and cognitive outcome 1 week after the end of the stimulation in one of the stimulation groups [161]. However, all other studies in this scope that investigated long-term changes after stimulation [159, 160, 165] found that the beneficial effects of the treatment were no longer apparent after longer observation periods, over which non-treated animals reached a similar level of recovery.

### Deep brain stimulation

With the possibility to target small and specific areas as well as deeper regions of the brain, DBS can be used to treat a wide variety of different impairments, such as the loss of cognitive [166–170] and motor function [171], as well as bladder dysfunction [172–174] and disorders of consciousness [175, 176]. Whilst the stimulation protocols differ greatly with respect to the targeted region and TBI sequelae, as shown in Table 3, the analyzed studies invariably reported positive results. Animals were generally kept awake during DBS, unless the stimulation was applied simultaneously with cystometric assessments [172–174]. Some studies utilized a current-controlled approach with amplitudes ranging from 20 to 200 µA [166–169, 175] or at 80% of the individual resting motor threshold [170, 171], while others applied voltages between 1 and 4 V [172–174, 176]. A stimulation frequency of 50 Hz seemed to be effective in the treatment of bladder dysfunction [172–174], while lower frequencies were used to treat motor [171] and cognitive deficits [166–168, 170], and higher frequencies of up to 200 Hz can be employed to increase arousal [175, 176]. Task-matched stimulation at 130 Hz for 5 s after each successful trial in a spatial learning test was also used to treat cognitive impairments after TBI [169]. In two studies, stimulation was applied directly before cognitive tests [166, 168], while, in the treatment of bladder dysfunction, stimulation was only triggered during cystometry when the measured bladder pressure exceeds a certain threshold [172–174]. Three studies applied stimulation over 12 daylight hours over several consecutive days to improve spatial memory [167, 170, 171], and two others investigating the potential of DBS to increase arousal started their continuous stimulation protocols directly after TBI over the course of 2 h to 1 day [175, 176]. The targeted brain area and stimulation onset highly depend on the treatment application in question, since DBS can be used to stimulate relatively small brain regions—compared to other stimulation methods—without affecting the surrounding tissue. Long-lasting effects of DBS were only reported in [169], where researchers observed improved recovery of spatial memory 10 days after cessation of stimulation compared to untreated animals; meanwhile, other studies reported that they did not find persisting effects on hippocampal theta power after stimulation was terminated [166, 168].

### Vagus nerve stimulation

VNS has been used in the preclinical studies listed in Table 4 to improve motor and cognitive impairments [177–179] as well as disorders of consciousness [180, 181] after TBI, but also in the treatment of cerebral edema [182, 183] and to prevent cell death [184]. Animals were usually awake during VNS, except in two studies where researchers intentionally anesthetized animals to investigate the effect of VNS on disorders of consciousness [180, 181]. One study does not state clearly whether animals were anesthetized during the VNS or not [183]. Four studies applied stimuli at an amplitude of 0.5 mA and a frequency of 20 Hz [177, 178, 182, 184], while three other studies used currents between 0.8 and 1 mA with a frequency of 30 Hz [179–181, 183], all of which chose to stimulate the left vagus nerve at the cervical level. Stimulation was often applied for 30 s in 30 min intervals over a period of up to 2 weeks, starting within 2 [177, 182] or 24 h after injury [178, 184], while two studies applied the stimulation only once, directly after induction of TBI [180, 181]. In one of the studies, stimulation was applied for 500 ms within 45 ms after each successful trial in a pull performance task, with the aim to improve motor function [179]. Only in one study stimulation was applied to the right vagus nerve at a frequency of 5 Hz with 5 ms pulses and an amplitude of 10 V, once for 20 min, in an effort to alleviate brain edema [183]. Most of the studies in this scope did not investigate any possible persisting effects, since VNS is mostly used as a continuous treatment after injury. The study conducted by Pruitt et al. measured persisting effects 1 week after the completion of VNS treatment; nevertheless, animals underwent further rehabilitation [179]. Two studies each observed that VNS attenuated the development of brain edema [182, 183], that it is effective for the treatment of cognitive [177, 178] or motor impairments [178, 179], had neuroprotective effects [183, 184], and promoted wakefulness after TBI [180, 181].

## Discussion

### Transcranial magnetic stimulation

Experiments with TMS in preclinical models of TBI attracted interest rather recently with the oldest study dating back to 2015. All of the analyzed TMS studies in this scope employ rTMS protocols for effective treatment. Given that the early phases after TBI are associated with cortical hypoexcitability [185, 186], high frequency rTMS has been the major focus of interest in the studied publications. This is in line with the treatment window in these studies, which often starts relatively soon after TBI. On the other hand, low frequency rTMS induces inhibitory effects, rendering neurons less likely to fire [82], and is mostly utilized in epilepsy research [187]. It should be noted that post-TBI hyperexcitability is also observed, though after some time with an onset after approximately 2 months in preclinical models [188] and it is associated with trauma-induced epilepsy. Notwithstanding, preclinical experiments with low frequency rTMS for the prevention of TBI-induced epileptogenesis are currently quite underrated and further research is needed.

The inclusion of appropriate control groups in TMS studies deserves critical emphasis. Verdugo-Diaz et al., for instance, showed that movement restriction alone, which is necessary for stimulation in awake animals, significantly reduced post-traumatic bleeding and mortality, and improved neurobehavioral scores to the same extent observed in the rTMS group [157]. Similarly, combination of rTMS with environmental enrichment (EE) reportedly led to improvements in sensorimotor function lasting up to 6 weeks compared to the rTMS alone [154]. However, in this study rTMS was applied for only 1 week post-TBI, whilst EE lasted for 6 weeks. Unfortunately, both untreated TBI and TBI + EE controls were not included in the beamwalk tests, leaving the question unanswered whether rTMS itself had any long-term contributions to the observed improvement.

Large variabilities in the used stimulation frequencies (2–40 Hz), stimulation durations (3–20 min), treatment periods (a few days to 4 weeks), as well as heterogeneity in the used protocols for pulse trains, make a direct comparison between these studies difficult. Stimulation parameters were either taken from previous studies investigating modes of injury other than TBI [152, 155–158], from clinical studies [153], or the choice of parameters was not mentioned [138, 154]. No two studies utilize comparable stimulation intensities, thus, a correlation of the stimulation parameters to different outcomes is hindered. Nevertheless, several studies with different TBI models, namely weight drop and controlled cortical impact (CCI), showed functional improvements upon rTMS starting 1 or 2 days after TBI, [138, 154–157] with daily sessions usually administered for 1 week or longer. However, in a rat model of lateral fluid percussion injury (FPI), rTMS starting 4 days after induction of severe TBI did not show any improvements in motor behavioral outcome [152], whilst in a CCI model of pediatric TBI beneficial effects were reported after starting rTMS 9 days post-injury [153]. Similar improvements in neurological scores were also reported after moderate TBI using Feeney’s weight drop model, when rTMS was started 4 days post-injury [158]. Reported cellular and molecular biological readouts suggest that the observed functional improvements could be the result of neuroprotection, thus a critical time window for the treatment after TBI can be presumed. However, the existence of such a therapeutic window, and whether it is influenced by factors such as age, gender, and trauma severity, is unclear due to the limited number of published preclinical studies on this topic as well as the large variability in used parameters and treatment regimens.

Biological correlates of observed functional improvements could include mitigation of apoptotic signaling and cell death [152, 156], as well as reduced loss of mature neurons [155, 156] and astroglial activation [155] together with increases in cell proliferation and neurogenesis in the neurogenic niches such as the subventricular zone of lateral ventricles [156]. Moreover, upregulations in the expression levels of brain-derived neurotrophic factor (BDNF), tropomyosin receptor kinase B (TrkB, neurotrophin receptor), *N*-methyl-d-aspartate receptor 1 (NMDAR1, glutamate receptor) and phosphorylation of cyclic AMP response element binding protein (CREB; induced by neuronal activation) [158] support the presumption that restoration of cortical excitability early after TBI has a critical role not only in attenuation of delayed loss of cells that survived the primary impact, but also in the enhancement of regenerative responses. These results are of peculiar importance for a better understanding of underlying biological correlates of improvements that were detected in clinical applications, as most of these readouts are devoid of any possibility of direct assessment in the clinical practice. Whilst the positive results are per se encouraging—despite large variabilities in injury type, trauma severity and stimulation parameters—the translational value of preclinical studies is invariably dependent on their power in delineating correlative and causative relations between the applied stimulation parameters and observed biological readouts. Therefore, maturation of preclinical research on post-TBI rTMS from the current exploratory phase towards standardized procedures that allow for systematic comparisons is highly desirable.

### Transcranial direct current stimulation

Similar to TMS, tDCS has only been under investigation in preclinical studies as a possible treatment for TBI sequelae in recent years, starting in 2016. Several of the selected studies investigated the same TBI sequelae and how tDCS could be used to treat them in a preclinical setting. Two studies from the same research group assessed the effect of tDCS on microvascular cerebral blood flow (mCBF), starting in the chronic phase 3 weeks after trauma induction using either repeated or single stimulation sessions [161, 164]. They could observe a restoration of impaired cerebrovascular reactivity to hypercapnia, improved cerebral blood flow and tissue oxygenation, which is a key factor in brain metabolism associated with brain damage in the acute phase. A decrease in blood flow regulation together with decreased tissue oxygenation is suspected to cause damage in the early phase post-injury. Moreover, a chronic reduction of local brain perfusion in patients with TBI is known to cause persisting effects on brain function [189] and is thus suspected to play a crucial role in long-term outcome. An improvement in motor function and excitability could be observed in response to a single tDCS session directly after TBI induction [160] or in the subacute phase 24 h after the injury [163]. The beneficial effect of the stimulation was apparent from the day after the stimulation in both experiments and up to 12 days later, where the experiment ended. In two other studies, the improvement in motor function in response to repeated tDCS over 4 weeks in the chronic phase was examined. The first of these studies, where stimulation was started 3 days after trauma, observed a significant difference to the sham-stimulation group from day 8 to day 26 post-injury [162]. In the second study, two groups with different time points of stimulation onsets, 1 and 3 weeks after injury, were compared [161]. The group with later onset of stimulation made a better recovery than when stimulation started 1 week after TBI, showing that tDCS led to a significant decrease in neurologic impairment and an increase in motor function, memory and learning. This finding was in part supported by another study, where tDCS was started either 1 or 2 weeks post-injury and lasted for 5 consecutive days [159]. Here, the results of the Rotarod test in the 2-week group were slightly better than in the 1-week group. However, the observed improvement in spatial memory was comparable in both groups. Long-lasting or persisting effects after the end of stimulation were assessed in four studies. The first showed a beneficial effect on motor function and spatial learning directly after tDCS sessions ended, however, 2 weeks later the animals in the other groups had recovered to a similar level [159]. The second study could show a persisting effect of the stimulation 1 week after the end of the treatment [161]. The third study investigated structural brain damage in MRI 12 days after the trauma immediately after tDCS, but did not find any significant volumetric changes such as hydrocephalus or cortical thinning in either of the groups (sham, repetitive mTBI, and repetitive mTBI with tDCS). Immunohistochemistry did not show any evidence of neuronal degeneration in sham, TBI or stimulated group. Immunohistochemical study with glial fibrillary acidic protein (GFAP) stain showed a slight hypertrophy of cell bodies and a minimal extension of cell processes in both the TBI and anodal tDCS group compared to the sham group 12 days after the trauma [160]. Another study, where stimulation was applied for 7 days starting 6 weeks after TBI, found no persisting effects after stimulation was stopped [165].

These findings lead to the conclusion that tDCS decreases the time needed for recovery. From the evidence presented above, it is unclear if tDCS is able to induce persisting changes in neuronal tissue, although an improvement of motor function and tissue oxygenation could be observed over several weeks. The effect of anesthesia on the treatment outcome is not apparent in the selected studies and the choice of anesthetizing animals during stimulation is not directly correlated to the impairment under investigation. Most studies adopted their stimulation parameters from research papers that treated impairments caused by something other than TBI [159, 163, 165] and two gave no specific reasoning for their choice of stimulation parameters [160, 161] and later reused them in publications for further investigations [163, 164].

Follow-up studies could focus on investigating changes to the established stimulation protocols and how these changes affect treatment outcome, while using electrodes with standardized surface areas or adjusting the amplitude of applied currents to reach comparable current densities. The timing of stimulation onset seems to be an important factor for a better treatment outcome, however, there are no commonalities concerning the optimal time point for the start of stimulation post-injury among these studies. Cathodal stimulation is rarely used in tDCS studies, even though it was shown to be an effective treatment to decrease impulsivity and increase attention after TBI [165], and there may be additional applications for it. Most of the studies in this scope assessed the histologic changes after TBI and tDCS treatment, which could serve as a solid basis for further research into the therapeutic mechanisms of tDCS.

### Deep brain stimulation

DBS first started to find use in preclinical studies about TBI treatment in 2013. The studies selected for this review used widely different stimulation protocols and time frames for each potential treatment application, which makes a comparison between them difficult. Almost half of preclinical DBS studies applied electrical stimuli continuously for 2 h [176], 1 day [175], or several weeks [167, 170, 171]. One research group initiated DBS whenever a signal measured via external urethral sphincter electromyography exceeded a certain threshold, in an attempt to enhance voiding efficiency [172–174]. Another group started stimulation directly before an experimental task in order to improve cognitive outcome [166, 168], while in one study stimulation was triggered every time a rodent successfully found a hidden platform in a Morris water maze test, with the goal to reinforce learning [169]. Most of the time, animals received stimulation in multiple sessions over several days [166, 168, 169, 175] or weeks [167, 170, 171], with others only applying a single session before the animals were sacrificed for further analysis [172–174, 176]. For the treatment of decreased arousal and disorders of consciousness, stimulation was usually initiated shortly after injury [167, 176], while treatment of bladder dysfunction started 1 week after induction of TBI [172–174]. Therapy of cognitive deficits was shown to be effective in the acute [167], subacute [166, 168] and chronic phases of TBI [169, 170].

Two studies used higher frequency stimulation of 100 Hz or more in the thalamic region to increase excitability in animals suffering from decreased arousal [175] or disorders of consciousness [176]. Stimulation frequencies as low as 7.7–8 Hz were applied in the midbrain or medial septal nucleus to treat cognitive deficits [166–168], while 30 Hz stimulation in the lateral cerebellar nucleus was used for a similar purpose [170, 171]. All three studies investigating DBS as a treatment for bladder dysfunction in this scope originate from the same research group and used identical stimulation parameters [172–174]. Their triggered approach consists of 10 s of 50 Hz stimulation at amplitudes between 1 and 2.5 V. In their most recent study [174], they explored simulation of the pedunculopontine tegmental nucleus instead of the rostral pontine reticular nucleus to investigate its neural connectivity with bladder function, resulting in a similar outcome. Aronson et al. applied 130 Hz biphasic pulses in trains of 500 ms in the nucleus accumbens, whenever an animal succeeded a given task, leading to an improved spatial memory in TBI rats [169]. Only one group reported that they found no beneficial effects after stimulating the medial septal nucleus at a frequency of 100 Hz [168]. While one study did undocumented preliminary research to find optimal stimulation parameters [166], others adopted their parameters from previous studies on different topics [171, 172, 175, 176] or made the selection and optimization of the stimulation protocols part of their study [167, 168, 173, 174]. Jen et al. found an ideal stimulus length and frequency for effective stimulation for their purpose [172], only to continue with investigations regarding the optimal stimulation intensity in further studies [173, 174]. Only Aronson et al. do not describe how they chose the exact stimulation parameters they use, but mention that phasic stimulation in the nucleus accumbens might be able to promote neural plasticity [169].

Three studies found that DBS in various locations can be used to improve motor function after TBI [167, 171, 175] and three others observed an improvement in voiding efficiency [172–174]. Two studies found that DBS improved spatial working memory [166, 167] and attenuated hippocampal theta activity [166, 168]. In one study, researchers observed a mediation of anti-apoptotic and anti-inflammatory effects after DBS [171], while another confirmed that it may promote wakefulness [176]. Most studies did not investigate any persisting effects of DBS. However, one study observed that the beneficial effects of their task-matched stimulation approach on spatial memory persisted 10 days after stimulation cessation [169], and several clinical studies have shown before that DBS leads to long-lasting positive changes in connectivity [190–192]. Animals were usually awake during DBS, except in studies involving cystometric measurements where they were anesthetized [172–174].

Researchers should continue building upon the insights gained in these studies about DBS as an effective preclinical treatment for TBI sequelae to find out more about the underlying mechanisms pertaining to precise electrical stimulation of specific brain areas. It would be desirable to find a consensus about the most effective stimulation parameters and time frames for a variety of impairments by comparing the effects of small parameter changes, as it was already shown in some studies in this scope. Experiments often lasted for less than 1 week, and animals were often sacrificed directly after an experiment or shortly after stimulation was terminated, having left no room for investigations into possible long-term improvements. Since DBS is used as a long-term treatment in clinical studies [114], preclinical studies should also address the effects of long-term stimulation. It remains to be seen if different impairments with related underlying neurologic causes may be treatable with similar stimulation protocols by stimulating in different brain regions.

### Vagus nerve stimulation

Compared to other stimulation modalities, the efficacy of VNS in the preclinical treatment of TBI sequelae has been investigated for a much longer time since 2005. Almost all VNS studies in this scope stimulated the left vagus nerve at the cervical level, except for one that targeted the right vagus nerve [183]. This consensus may stem from the fact that the right vagus nerve has more projections to the sinoatrial node of the cardiac atria and stimulation could therefore have an undesirable effect on the cardiac rhythm [130].

In the analyzed publications, most research was focused on treatment with multiple repeated stimulation sessions [177–179, 182, 184], while a few publications report the effects of single session VNS treatment [180, 181, 183]. The onset of the treatment in studies using repeated stimulation varied between 2 h [177, 182], 24 h [178, 184] and up to 9 days [179] after sustaining TBI. These time points correspond to different phases of post-injury pathology: early acute phase, subacute phase and chronic phase. In a clinical setup, therapy can be implemented at any point after TBI; however, early interventions are known to lead to better functional and psychological outcomes in patients [193–196]. Additionally, the long-term study of Pruitt et al. delivered stimuli within 45 ms after each successful pull trial [179], which should lead to strengthening of synaptic connections according to the STDP model of plasticity. In the studied publications, an early onset of the VNS treatment led to a faster recovery of motor skills, which is usually observed around day 2 [177, 182], as compared to a subacute onset from day 4 on [178]. Conversely, starting stimulation 24 h after TBI encouraged a faster improvement of cognitive functions; 13 days for early-onset [177] compared to 11 days for the later-onset study [178]. The study implementing VNS in the chronic phase also showed a positive effect of VNS on motor recovery [179]. However, it was sustained for 5 weeks and accompanied by physical training of the animals. Persisting effects of VNS were described for 1 week after cessation of the treatment. Multiple stimulation sessions also proved to have a neuroprotective effect on GABAergic neurons [184] and limit edema formation in the ipsilateral cortex [182]. In summary, repeated VNS aids in functional recovery after TBI and to some extent helps in constraining secondary damage.

Single stimulation after TBI led to a wake-promoting effect in free-fall injury animal models [180, 181] and the promotion of anti-inflammatory cytokine modulation with lower edema formation in a blast injury model [183]. This might indicate that an isolated VNS session could be advantageous in early post-injury stages and might lead to diminishing secondary injury. Nevertheless, clinical application of single VNS treatment would be plausible in the form of non-invasive stimulation, rather than during surgery. Transcutaneous VNS has already been proven feasible and was well tolerated in humans with severe TBI [197]. Pre-clinical studies employing this kind of VNS treatment for TBI are not available at this moment, but have been described for models of inflammation [198, 199], ischemia [200] and seizures [201].

Unlike in the case of TMS, the VNS studies in this scope use comparable stimulation protocols. Four publications coming from one research group report using the same stimulation parameters, which authors described that they were adapted from a previous study [177, 178, 182, 184]. This leads to a better reproducibility of the experiments and facilitates comparability of the results. Other studies mention implementing the same stimulation protocols as previous publications where the respective impairments had a different underlying cause than TBI [179–181], while one study does not mention how stimulation parameters were selected [183].

Since VNS is an established method and was FDA-approved for drug-resistant epilepsy and depression [202], there is an abundance of commercially available devices for human patients. However, similar devices for rats are currently not accessible and only some of the publications [177, 178, 182, 183] describe the electrodes they were implanting. Post-experimental re-testing of the electrodes is reported in only three of them [177, 178, 184]. None of the studies mentions pre-surgical evaluation of the devices, which might be crucial to ensure proper functionality. Similarly, observed side effects were also not reported in any of the analyzed publications, which could lead to insights into safety of VNS application in patients.

Since VNS is usually used as a long-term treatment in awake patients, the effect of anesthesia on the stimulation outcome is not investigated, unless it is specifically used as a treatment for disorders of consciousness [180, 181]. However, these studies report usage of a chloral hydrate, a drug considered not suitable for anesthesia of laboratory animals [203], and mention inducing anesthesia three times during 1 day in some of the experimental groups, which is a considerable burden for animals. Therefore, these results should be interpreted with caution.

Compared to the other stimulation methods presented above, there is more consensus between different VNS studies. This method proved to be advantageous for therapy of different conditions associated with TBI, regardless of the temporal window of its implementation and the amount of stimulation sessions. Further studies aimed at different modalities of VNS, e.g. transcutaneous VNS, and combination with other therapeutic agents, such as physiotherapy and pharmacotherapy, as well as life-long studies might lead to additional insights into better applications of VNS in humans.

### Comparison between different methods

All of the methods discussed here can be used to treat motor and cognitive dysfunctions and lead to significant improvements in TBI animal models [138, 153–155, 159, 160, 162, 163, 166–171, 177–179]. Only one study found that their TMS protocol did not induce any beneficial effects regarding motor improvements [152], which was likely due to the relatively short stimulation duration they used compared to other studies. At the same time, there are a variety of other TBI sequelae that benefit from treatment with different electrical stimulation modalities. Neuroprotective effects can be induced with TMS, DBS and VNS to prevent further cell-death after injury [153, 155, 171, 183, 184]. Both TMS and tDCS are able to modulate cortical excitability leading to plasticity and increased brain activity [153, 154, 160, 165]. Suppression of cortical excitability can be achieved with TMS and tDCS as well, leading to a decrease in hyperactivity and impulsivity in animals [153, 165]. After stimulation with TMS and DBS, researchers discovered beneficial changes in histological assessment [157, 158, 168–171], while some tDCS and VNS studies show positive effects on protein expressions after treatment [159, 160, 162, 180, 183]. Finally, the studies in the scope of this review show that tDCS, DBS and VNS may effectively be used to promote wakefulness and treat disorders of consciousness caused by TBI [160, 175, 176, 180, 181].

While these stimulation methods have many treatment opportunities in common, each of them have possible applications that have not yet been observed with the other modalities in TBI animal models, giving them a status as some sort of “specialization”. TMS has been used to improve brain metabolism and potentially induce cell proliferation and neurogenesis [156], while tDCS studies showed that it can be used to increase microvascular flow and tissue oxygenation [161, 164]. This likely stems from the fact that these two methods both activate large parts of the cortex, therefore having a higher impact on the metabolism and oxygenation of the brain. Exclusively, DBS studies explored the application of electrical stimulation to improve voiding efficiency in animals with bladder dysfunction [172–174], since DBS can be used to specifically target diseases whose etiology is connected to single brain regions. Only VNS, which is known to decrease the disruption of the blood–brain barrier [120], has been used in preclinical studies to attenuate the development of cerebral edema after TBI [182, 183].

### Translatability of the results

There are several aspects of pre-clinical studies that should be taken into consideration while analyzing their translatability into a clinical environment. Among them worth mentioning are: the relevance of the animal model, appropriate treatment, the temporal window, and side effects.

All of the analyzed studies were performed with well-established mammalian model species: rat, mouse and rabbit. The most frequently used model species was rat (28/34), with Sprague–Dawley as a leading strain (19/28), followed by Long Evans rats (7/28) and a single instance of Wistar rats used. A minor portion of analyzed studies was performed on mice (5/34) and only one publication reports experiments on New Zealand rabbits. The dominance of the rat model stems from a relatively big size of the brain in these animals, as compared to mice. This translates to convenience during surgery, especially when small electrodes are implanted, but is also important for a better spatial resolution when targeting specific brain regions [204], for instance with TMS. Common use of Sprague–Dawley rats ascertains comparability of the results within and between neurostimulation methods. However, Sprague–Dawley rats were reported to reach a faster motor skill recovery as compared to Long Evans rats [205]. Therefore, caution is recommended when comparing the two strains with each other. Moreover, all of those species are lissencephalic and display different geometry, craniospinal angle and grey-to-white matter ratio than humans [206], which is a further limitation of the translatability of results to human patients. Only one publication [153] used juvenile rats as a model for a TMS study. Since TBI is the disease with one of the highest incidences in children and youth below 19 years old [207], it is of utmost importance to further encourage studies employing neurostimulation methods as a post-traumatic therapy in young animals, with special focus on non-invasive methods.

Sex-dependent differences in the outcomes of TBI pre-clinical studies have been widely reported and reviewed in multiple studies [208–210]. In general, animal studies report better outcomes in females than in males, which might stem from the neuroprotective effects of estrogen and progesterone [208, 209]. The desire to determine treatment efficacy independent of hormonal status leads to the selective inclusion of males in pre-clinical studies, unless the study is specifically designed to address the sex difference itself [208, 209]. Likewise, only a small proportion of publications analyzed here reports use of female animals (2/34) [172, 179] or both sexes (2/34) [176, 180], which restrains the translatability of the results into human patients. Inclusion of female animals in experimental post-traumatic neurostimulation research is recommended for a better representation of the clinical situation.

Methods of inducing TBI varied: 15/34 studies used a weight-drop method, 10/34 fluid percussion injury, 8/34 controlled cortical impact and 1/34 performed blast injury. Except for blast injury, which is not fully consistent, these models are highly standardized and cover different types of injury, from focal to diffuse and mixed [206], corresponding to lesion diversity in patients who have survived head injury. The reported severity of the TBI model also varies: mild TBI was induced in 3/34 of studies, mild-moderate TBI in 2/34, moderate TBI in 13/34 and severe in 5/34. This does not fully mirror the clinical situation in humans, where approximately 80% of TBI is categorized as mild [211]. Nevertheless, moderate and severe TBI constitute approximately 50% of hospitalizations [212] and lead to higher mortality [213, 214]. Regrettably, a significant proportion of studies (11/34) does not report the severity level of the injury, substantially limiting their translatability. It is also worth noting that, due to anatomical and coil size differences, TMS may be able to stimulate deeper brain regions in small rodents that could otherwise not be effectively targeted in human patients [25].

Appropriate treatment requires a suitable method and stimulation protocol for the disability under investigation in the respective study. This is especially an issue for the clinical applicability of the TMS, tDCS and VNS studies in the scope of this review, since they use widely different stimulation parameters and time frames, even for the treatment of similar disabilities. In case of VNS, stimulation protocols were comparable; however, studies performing acute VNS intraoperatively might not be as clinically relevant.

The temporal window of applied stimulation methods varies highly. Early onset of the stimulation protocol was reported in 9/34 of publications analyzed, subacute in 13/34 and chronic in 13/34 of studies. Interestingly, individual methods seem to be applied at specific time points: TMS almost exclusively in the subacute stage, tDCS mostly in the subacute and chronic stages, and VNS in the acute and subacute stages, while only DBS finds application in all stages after TBI. This distribution of the time points may correspond well to the clinical situation, in which onset and duration of the therapy are highly variable [215–217].

Finally, possible adverse effects of the treatment are an important factor as well. The presence of side effects during pre-clinical studies might indicate plausible future problems in the clinical setting and should not be underestimated. Review articles on side effects caused by therapeutic application of TMS, tDCS and VNS in a clinical setting report only mild side effects [218–220], while adverse effects of DBS require more investigation in closer cooperation of scientists and clinicians [221] and are prone to bias [222]. Very few of the studies in this scope investigated possible side effects of any of these four stimulation methods and not a single one reported that they found any negative implications, which similarly hints to a possible bias and would be an important aspect in further research on this topic.

## Conclusion

This literature review was conducted in order to give a comprehensive overview on the most commonly applied electrical stimulation techniques used in conjunction with preclinical models to investigate their potential for rehabilitation after TBI. Our approach focused on the specific stimulation parameters and time frames used in the analyzed studies with the goal to help optimize treatment applications. One limitation of this review is the fact that it focuses specifically on the treatment of TBI sequelae, leaving it blind to stimulation protocols used for similar impairments with different underlying causes. Nevertheless, TBI treatment is one of the main applications for electrical stimulation paradigms, which is why this review showcases a large portion of the research conducted in this field.

We found that for some stimulation methods, specifically tDCS and VNS, researchers have started using comparable protocols over the recent years, increasing their focus on the specific cellular mechanisms leading to an improved outcome. TMS and DBS, however, are used for the treatment of a diverse group of TBI sequelae, employing widely different stimulation parameters and starting at various time points after injury. This makes it difficult to find optimal treatment solutions and leaves a lot of questions about further improvements that could be achieved through small adjustments to these parameters and time frames. Further research in this field should focus on building upon the insights documented in previous publications by using comparable experimental models and varying parameters such as stimulation frequencies, amplitudes, duration, onset after injury and how often it is repeated, while looking at cognitive and behavioral improvements, as well as beneficial changes occurring at the cellular level. Researchers should look at the long-term effects of electrical stimulation methods in TBI therapy, which were rarely investigated in the publications analyzed herein. However, it is clear that all four of the stimulation modalities in the focus of this review show promising results and have the potential to shape the future of clinical treatment of patients following TBI.

## Data Availability

All data generated or analyzed during this study are included in this published article. Datasets with more detailed information are available from the corresponding author on reasonable request.

## Abbreviations

BDNF: Brain-derived neurotrophic factor
CaMKII: Calcium/calmodulin-dependent protein kinase 2
cAMP: Cyclic adenosine monophosphate
CCI: Controlled cortical impact
CLOCK: Circadian locomotor output cycles protein kaput
CMG: Cystometrogram
CREB: CAMP response element-binding protein
CRY2: Cryptochrome 2
CST: Corticospinal tract
CT: Computed tomography
DBS: Deep brain stimulation
DoC: Disorders of consciousness
ECS: Electrical cortical stimulation
EE: Environmental enrichment
EEG: Electroencephalogram
EES: Epidural electrical stimulation
ELISA: Enzyme-linked immunoassay
ES: Electrical stimulation
EUS-EMG: External urethral sphincter electromyography
fMRI: Functional magnetic resonance imaging
FPI: Fluid percussion injury
GABA: Gamma-aminobutyric acid
GABABR: Gamma-aminobutyric acid beta receptor
GAD: Glutamic acid decarboxylase
GAP43: Growth associated protein 43
GFAP: Glial fibrillary acidic protein
IL-10: Interleukin-10
IL-1β: Interleukin-1 beta
LCN: Lateral cerebellar nucleus
LFMS: Low-field magnetic stimulation
LFP: Local field potential
LHA: Lateral hypothalamic area
LSCI: Laser speckle contrast imaging
LSM: Laser scanning microscopy
LTD: Long-term depression
LTP: Long-term potentiation
M1: Primary motor cortex
mCBF: Microvascular cerebral blood flow
MEP: Motor-evoked potential
MeSH: Medical subject headings
mNSS: Modified Neurological Severity Score
MR: Midbrain raphe
MRI: Magnetic resonance imaging
mRNA: Messenger RNA
MRS: Magnetic resonance spectroscopy
MSN: Medial septal nucleus
mTBI: Mild traumatic brain injury
MUA: Multi-unit activity
NADH: Nicotinamide adenine dinucleotide
NeuN: Neuronal nuclei
NMDA: *N*-Methyl-d-aspartate
NSS: Neurological severity screen
OX1R: Orexins receptor type 1
P-CREB: Phosphorylated CREB
p75NTR: P75 neurotrophin receptor
PCR: Polymerase chain reaction
PET: Positron emission tomography
PnO: Rostral pontine reticular nucleus
PPTg: Pedunculopontine tegmental nucleus
PrPc: Prion protein
PSD-95: Postsynaptic density protein 95
RMT: Resting motor threshold
rmTBI: Repetitive mild traumatic brain injury
RNA: Ribonucleic acid
RRT: Rotarod test
rTMS: Repetitive transcranial magnetic stimulation
RT-PCR: Reverse transcription PCR
SEP: Somatosensory-evoked potential
SPRT: Single-pellet reaching task
STDP: Spike timing-dependent plasticity
SVZ: Subventricular zone
SYN: Synaptophysin
TBI: Traumatic brain injury
tDCS: Transcranial direct current stimulation
TEM: Transmission electron microscopy
TMS: Transcranial magnetic stimulation
TNF-α: Tumor necrosis factor alpha
TrkB: Tropomyosin receptor kinase B
VNS: Vagus nerve stimulation
α1-AR: Alpha-1 adrenoceptor
